# Mineral Supplementation Increases Erythrose Reductase Activity in Erythritol Biosynthesis from Glycerol by *Yarrowia lipolytica*

**DOI:** 10.1007/s12010-014-0745-1

**Published:** 2014-02-01

**Authors:** Ludwika Tomaszewska, Waldemar Rymowicz, Anita Rywińska

**Affiliations:** Department of Biotechnology and Food Microbiology, Wrocław University of Environmental and Life Sciences, Chełmońskiego Str. 37/41, 51-630 Wrocław, Poland

**Keywords:** Erythritol, Erythrose reductase, Glycerol, Minerals, *Yarrowia lipolytica*

## Abstract

The aim of this study was to examine the impact of divalent copper, iron, manganese, and zinc ions on the production of erythritol from glycerol by *Yarrowia lipolytica* and their effect on the activity of erythrose reductase. No inhibitory effect of the examined minerals on yeast growth was observed in the study. Supplementation with MnSO_4_·7H_2_O (25 mg l^−1^) increased erythritol production by *Y. lipolytica* by 14.5 %. In the bioreactor culture with manganese ion addition, 47.1 g l^−1^ of erythritol was produced from 100.0 g l^−1^ of glycerol, which corresponded to volumetric productivity of 0.87 g l^−1^ h^−1^. The addition of Mn^2+^ enhanced the intracellular activity of erythrose reductase up to 24.9 U g^−1^ of dry weight of biomass (DW), hence, about 1.3 times more than in the control.

## Introduction

Erythritol is a sugar alcohol used as a natural and safe low-calorie sweetener (0–0.2 kcal g^−1^). In comparison to sucrose, it has 60–70 % of its sweetness, a similar taste profile, and rheological properties. Among other sugar substitutes, it is free of undesired aftertaste, tooth-friendly, safe for diabetics, and does not induce gastric distress [1–3]. It is the only sugar alcohol produced in biotechnological processes, as its chemical production is ineffective. The capability for its production was reported for osmophilic yeast, some fungi, and bacteria. Traditional substrates used for the biosynthesis include glucose, fructose, sucrose, and starch hydrolysates [3, 4]. However, the food market’s demand for erythritol aroused increased attention in ways to reduce the cost of erythritol production through screening the high-yield producing strains and adjusting process conditions. Many efforts have been focused, therefore, on evaluating the impact of factors such as temperature, pH, osmotic pressure, carbon and nitrogen sources, substrate concentration, addition of NaCl/KCl, and supplementation with vitamins and minerals [5–11].

In the last decade, much attention was paid to the possibility of applying industrial and agroindustrial by-products and wastes as low-cost substrates for microbiological processes. Recent trends to intensify biofuel production have resulted in high quantities of raw glycerol, a by-product of biodiesel industry, appearing on the market. The valorization of glycerol focused especially on its conversion into 1,3-propanediol, dihydroxyacetone, ethanol, organic acids, and single-cell oil [12–15]. It is worthy of notice that in this field much attention was devoted to the biotechnological potential of an unconventional yeast *Yarrowia lipolytica*. It was demonstrated that efficient production of biomass, organic acids, polyols, and lipid synthesis might be obtained when the biosynthesis was performed in pure and raw glycerol-containing media, which has been already well-described [15, 16]. The conversion of glycerol into erythritol by *Y. lipolytica* seems to be especially interesting [17–19].

The main factor in erythritol biosynthesis from glycerol by *Y. lipolytica* is reduced pH of the culture medium. However, the increased osmotic pressure of the environment seems to be important as well [20]. Although erythritol production by *Y. lipolytica* has been extensively reported in the literature, it has, so far, never been studied as being affected by the addition of minerals.

The presented study investigated the effect of divalent copper, iron, manganese, and zinc ions on erythritol production from glycerol and their impact on the activity of erythrose reductase during erythritol biosynthesis by *Y. lipolytica*.

## Materials and Methods

### Microorganism

An acetate-negative mutant strain Wratislavia K1 of *Y. lipolytica* [21] was used in this study.

### Media

**Fig. 1:**
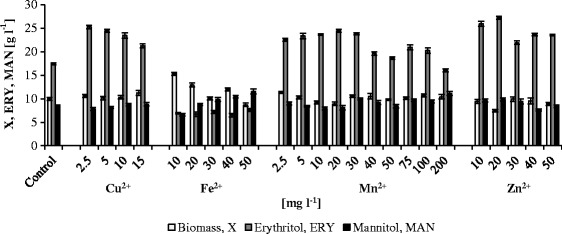
Biosynthesis of erythritol from glycerol in the shake-flask experiment by *Y. lipolytica* Wratislavia K1 in the presence of different minerals in the medium

**Fig. 2:**
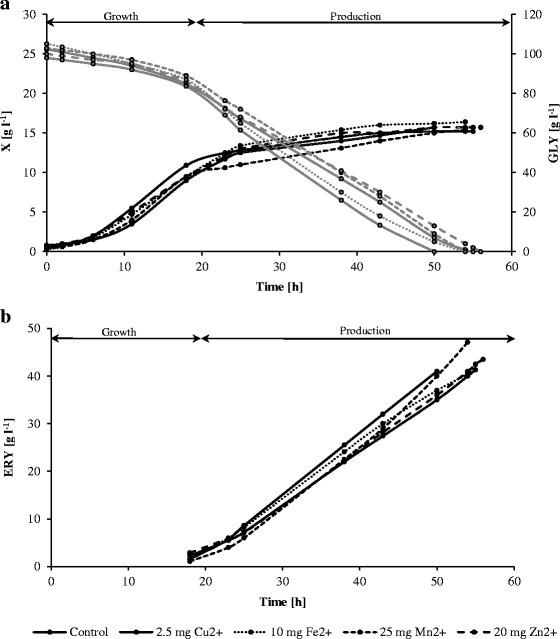
**a** Yeast growth (*black curves*) and glycerol consumption (*gray curves*) during erythritol formation by *Y. lipolytica* in the cultures without and with minerals: 2.5 mg l^−1^ of Cu^2+^, 10 mg l^−1^ of Fe^2+^, 25 mg l^−1^ of Mn^2+^, and 20 mg l^−1^ of Zn^2+^. **b** Erythritol production (*black curves*) by *Y. lipolytica* Wratislavia K1 in the cultures without and with mineral additions

Inoculation medium contained the following (g l^−1^): glycerol (98 %, w/w) — 50.0; yeast extract — 3.0; malt extract — 3.0; bacto-peptone — 5.0; and distilled water — 1 l. Production medium for shake-flask experiment consisted of the following (g l^−1^): glycerol — 100.0; (NH_4_)_2_SO_4_ — 2.5; MgSO_4_·7H_2_O — 1.0; yeast extract — 1.0; KH_2_PO_4_ — 0.2; CaCO_3_ — 3.0; distilled water — 1 l; and minerals: FeSO_4_·7H_2_O, MnSO_4_·7H_2_O, CuS0_4_·5H_2_O, and ZnSO_4_·7H_2_O in concentrations presented in Fig. 1, pH 3.0. For bioreactor cultures, the production medium was prepared from the following (g l^−1^): glycerol — 100.0; (NH_4_)_2_SO_4_ — 2.25; MgSO_4_·7H_2_O — 1.0; KH_2_PO_4_ — 0.22; yeast extract — 1.0; NaCl — 26.5; distilled water — 1 l; and minerals. For minerals used, see Fig. 2 or Table 1. All the media were sterilized at 121 °C for 20 min.

**Table 1:** Effect of minerals on the production parameters and the activity of erythrose reductase during erythritol biosynthesis from glycerol by *Y. lipolytica* Wratislavia K1

Cultures	Parameters							Erythrose reductase [U g^−1^ of DW]
	Time^*^ [h]	X	ERY [g l^−1^]	MAN	Y_ERY_ [g g^−1^]	Q_ERY_ [g l^−1^ h^−1^]	q_ERY_ [g g^−1^ h^−1^]	
Control	55 ± 2^b^	15.2 ± 1.8^a^	41.3 ± 2.3^a^	2.1 ± 0.3^a^	0.40 ± 0.03^a^	0.75 ± 0.07^a^	0.049 ± 0.002^a^	18.5 ± 0.2^a^
2.5 mg l^−1^ Cu^2+^	50 ± 2^a^	15.7 ± 1.7^a^	41.0 ± 1.5^a^	2.9 ± 0.7^ab^	0.41 ± 0.02^a^	0.82 ± 0.02^ab^	0.052 ± 0.006^a^	22.7 ± 0.3^c^
10 mg l^−1^ Fe^2+^	54 ± 3^ab^	16.4 ± 2.2^a^	42.4 ± 2.5^a^	4.3 ± 1.2^b^	0.40 ± 0.02^a^	0.79 ± 0.01^a^	0.048 ± 0.006^a^	20.9 ± 0.4^b^
25 mg l^−1^ Mn^2+^	54 ± 2^ab^	15.4 ± 1.9^a^	47.1 ± 1.9^b^	1.8 ± 0.4^a^	0.47 ± 0.01^b^	0.87 ± 0.07^b^	0.056 ± 0.011^a^	24.9 ± 0.3^d^
20 mg l^−1^ Zn^2+^	56 ± 3^b^	15.9 ± 3.1^a^	43.5 ± 3.1^ab^	1.9 ± 0.9^a^	0.43 ± 0.02^a^	0.77 ± 0.02^a^	0.048 ± 0.007^a^	23.0 ± 0.5^c^

^*^Means in the same column with different letters (a, b, c, and d) are significantly different; P ≤ 0.05

### Culture Conditions

Growth culture was carried out in 0.3-l flasks containing 0.05 l of the inoculation medium on a rotary shaker (CERTOMAT IS, Sartorius Stedim Biotech) at 29.5 °C and 140 rpm for 72 h. Inocula of 1.0 ml and 0.2 l were used to inoculate flask and bioreactor cultures, respectively. The shake-flask experiment was conducted for 7 days in 0.3-l flasks containing 0.03 l of the production medium for shake-flask experiment, under the same conditions as described above. At the end of the culture, CaCO_3_ was neutralized by HCl (1:1), and the final volume of the culture broth was adjusted to its initial volume with distilled water, before analysis. Bioreactor fermentations were performed in a 5-l stirred-tank reactor (BIOSTAT B-PLUS; Sartorius, Germany) with a 2.0-l working volume at 29.5 °C, 800 rpm, 0.6 vvm, and pH 3.0, maintained automatically by the addition of NaOH (20 %, w/v). The samples were taken two or three times a day. All fermentations were performed in triplicate, and the results were presented as mean values.

### Analytical Methods

The dry cell weight, substrate, product, and by-products concentrations were determined according to the earlier-described method [20].

### Enzyme Assay

The activity of erythrose reductase was examined after 24 h of cultivation. The sample of 0.2 l was harvested from the bioreactor culture by centrifugation (10 min, 4 °C, 4,300 ×*g*). The biomass was washed twice with a 0.1 M phosphate buffer (pH 7.4) and centrifuged. Next, the biomass was resuspended in 0.05 l of a disruption buffer (1 mM EDTA, 5 mM PMSF, 5 mM DTT, and 0.1 M phosphate buffer pH 7.4) and disrupted with glass beads using Sonics VCX500 (30 min, 4 °C). After the sonification, the sample was centrifuged (20 min, 4 °C, 9,800 ×*g*), and erythrose reductase (EC 1.1.1.21) activity was determined in the supernatant according to Lee et al. [22]. The protein was assayed with Lowry’s method. One unit of the activity (U) represents 1 μmol of NADPH consumed per 1 min (λ = 340 nm) at 22 °C. The enzyme activity was expressed as U per gram (g) of yeast biomass dry weight (U g^−1^of DW).

### Statistical Analysis

Results of the study were analyzed statistically using Statistica 10.0 software (StatSoft, Tulsa, USA). One-way analysis of variance was performed to detect significant differences in the data, depending on the minerals’ presence in the media. Homogenous groups were determined using Duncan’s test (P ≤ 0.05).

## Results and Discussion

### Effect of Minerals on Yeast Growth and Erythritol Production

The effect of supplemental Cu^2+^, Fe^2+^, Mn^2+^, and Zn^2+^ on erythritol biosynthesis by Wratislavia K1 strain of *Y. lipolytica* was first examined in the 7-day shake-flask cultures (Fig. 1). The metal ions were added in the form of inorganic salts: CuSO_4_·5H_2_O, FeSO_4_·7H_2_O, MnSO_4_·7H_2_O, and ZnSO_4_·7H_2_O. In the control culture without minerals, concentrations of biomass, erythritol, and mannitol after cultivation reached 9.9, 17.4, and 8.4 g l^−1^, respectively.

Copper ions were applied in the concentrations of 2.5–15.0 mg l^−1^. At the end of the experiment, glycerol was exhausted and biomass concentration ranged from 10.1 to 11.2 g l^−1^ in all the culture broths. Cu^2+^ significantly improved erythritol biosynthesis—especially when added in the concentrations of 2.5 and 5.0 mg l^−1^ which assured erythritol production at 25.2 and 24.4 g l^−1^, respectively. In all experimental variants, the concentration of mannitol was similar to that in the control culture.

It was found that iron ions increased biomass concentration when added in the concentrations under examination (10.0–50.0 mg l^−1^), although in these cultures glycerol was not totally consumed and its residual concentration varied from 10.1 to 15.3 g l^−1^ (data not presented). Shake-flask cultures supplementation with Fe^2+^ significantly inhibited erythritol formation to the level of 6.9–7.6 g l^−1^. Generally, mannitol concentration was slightly improved by ion addition and, at 50.0 mg l^−1^ of FeSO_4_·7H_2_O, its concentration reached 11.5 g l^−1^.

Manganese ions were applied in a wide range of concentrations, i.e., 2.5–200.0 mg l^−1^. In the cultures with more than 40.0 mg l^−1^ of MnSO_4_·7H_2_O, the residual glycerol concentration amounted to 4.5 g l^−1^. The ion addition did not affect yeast growth significantly, as biomass level varied from 8.9 to 11.3 g l^−1^. Mn^2+^ was observed to have a great positive impact on erythritol formation. When cultures were supplemented with 2.5–30.0 g l^−1^ of MnSO_4_·7H_2_O, erythritol concentration was in the range of 22.5–24.4 g l^−1^. Mannitol concentration was slightly increased by manganese ions and reached up to 11.1 g l^−1^.

In the cultures with zinc ions applied in the concentration of 10.0–50.0 mg l^−1^, glycerol was completely consumed at the end of the experiment. Erythritol biosynthesis was significantly enhanced by the addition of Zn^2+^ and erythritol level varied from 20.9 to 27.2 g l^−1^. The best results were obtained when the culture was supplemented with 20.0 mg l^−1^ of ZnSO_4_·7H_2_O. Mannitol formation was improved as well but only to a minor extent.

The presence of other by-products, i.e., arabitol, citric, and α-ketoglutaric acids, was detected in all the cultures (data not presented). The highest concentration of arabitol was observed in the cultures with Fe^2+^, but the polyol concentration did not exceed 2.9 g l^−1^. In the cultures supplemented with Cu^2+^ and Mn^2+^, the assays showed the highest amounts of citric (5.0 g l^−1^) and α-ketoglutaric (2.0 g l^−1^) acids, respectively.

To date, the effect of different metal ions on erythritol production has been studied in shake-flask cultures in glucose media [8, 11]. Among all examined minerals, the stimulatory effect was observed only when Cu^2+^ and Mn^2+^ were present in the cultures with *Torula* sp. [8]. Generally, the addition of Ca^2+^, Fe^2+^, and Zn^2+^ did not affect the process, whereas the presence of Cr^2+^, Ni^2+^, and V^2+^ inhibited erythritol production when added in the concentrations higher than 20.0 mg l^−1^. In turn, in the erythritol biosynthesis by *Candida magnoliae*, the addition of Zn^2+^, Fe^2+^, and Ca^2+^ resulted in a slight increase of erythritol concentration, while supplementation with Cu^2+^ and Mn^2+^ inhibited erythritol formation [11]. Additionally, it was also reported that copper ions caused a reduction of other polyols, whereas manganese, iron, and zinc ion supplementation resulted in increased amounts of by-products.

### Increased Erythritol Production with Mineral Supplementation in Bioreactor Batch Cultures

For more in-depth investigation of the minerals’ effect on erythritol biosynthesis, they were then added to the bioreactor cultures (Fig. 2, Table 1). The medium enriched with NaCl and pH 3.0 was used for these cultures, as our previous research demonstrated that salt and reduced pH value significantly improved erythritol production yield and its volumetric productivity [17, 20]. All the processes were run until the complete exhaustion of glycerol. In the control culture without minerals, after 55 h, the concentration of biomass reached 15.2 g l^−1^ and that of erythritol amounted to 41.3 g l^−1^, which corresponded to a yield of 0.40 g g^−1^ and volumetric productivity of 0.75 g l^−1^ h^−1^.

In the processes with minerals, biomass concentration remained at a comparable level (15.2–16.4 g l^−1^). In comparison to the control, a similar concentration of erythritol and shorter biosynthesis time were obtained in the culture with Cu^2+^, which resulted in the improved value of volumetric productivity (0.82 g l^−1^ h^−1^). Slightly increased amounts of erythritol were observed in the cultures with Fe^2+^ and Zn^2+^, but the yield and biosynthesis parameters were comparable to the control. The best results were obtained when 25.0 mg l^−1^ of MnSO_4_·7H_2_O was added to the culture, where 47.1 g l^−1^ of erythritol was produced with a yield of 0.47 g g^−1^, volumetric productivity of 0.87 g l^−1^ h^−1^, and specific production rate of 0.056 g g^−1^ h^−1^.

The concentration of by-products in bioreactor processes was significantly decreased in comparison to shake-flask cultures. The highest concentration of mannitol reached 4.3 g l^−1^ in the culture with Fe^2+^, whereas, in other cultures, it ranged from 1.8 to 2.9 g l^−1^ (Table 1). The amounts of arabitol, citric, and α-ketoglutaric acids were relatively low and did not exceed 1.2 g l^−1^ (data not presented).

The tolerance to an increased concentration of minerals and trace elements in the culture medium is an important feature of the production strains when process industrialization is considered. On the basis of the presented results, no inhibitory effect on yeast growth was observed in the cultures with ions addition. Hence, it was found that Wratislavia K1 strain was characterized by a high tolerance to the examined minerals. Literature reports confirmed that different yeasts, including *Y. lipolytica*, have a high tolerance to metal ions and abilities to capture and accumulate their high concentrations in cells [23, 24]. Moreover, minerals were reported to affect the production of several polyols [8, 11, 25, 26]. For example, in the bioreactor cultures of *Torula* sp. yeast produced 155.0 g l^−1^ of erythritol from 400.0 g l^−1^ of glucose [8]. The addition of Mn^2+^ and Cu^2+^ allowed to increase the erythritol concentration as well as the values of biosynthesis parameters such as yield and volumetric productivity, similarly as it was observed in the presented work. The best results of the biosynthesis were obtained upon the coupled addition of Mn^2+^ and Cu^2+^ ions, which resulted in erythritol production at 196.0 g l^−1^ with a volumetric productivity of 1.45 g l^−1^ h^−1^. The erythritol production yield was improved to 48.9 %; therefore, it was comparable to erythritol yield (47.1 %) obtained from glycerol in this study.

The impact of minerals on the production of other *Y. lipolytica* metabolites was as well described in the literature. The presence of copper, iron, manganese, and zinc had a positive effect on yeast growth when cultivated on glucose [27]. However, only the presence of Zn^2+^ ions improved the biosynthesis of citric acid from glucose. The addition of Mn^2+^ decreased citric acid production, whereas Cu^2+^ and Fe^2+^ inhibited formation of this metabolite. When grown on ethanol-containing media, *Y. lipolytica* showed increased demand for zinc ions [28]. Moreover, the enhanced effect of zinc and iron ions was reported on the conversion of ethanol into citric, isocitric, and α-ketoglutaric acids [28–30]. High requirements of iron in the cells of *Y. lipolytica* were also necessary for the effective biosynthesis of isocitric acid when yeasts were cultivated in rapeseed oil media [31].

### Effect of Minerals on Erythrose Reductase Activity

**Fig. 3:**
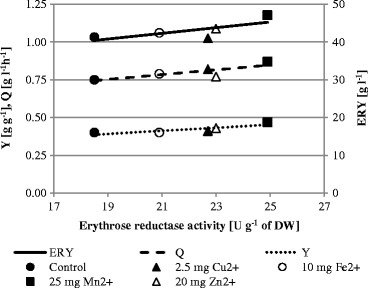
Correlations of erythrose reductase activity with erythritol biosynthesis parameters (erythritol concentration, ERY; volumetric productivity, Q; and erythritol production yield, Y) obtained in the cultures with different mineral additions

To investigate the roles of Cu^2+^, Fe^2+^, Mn^2+^, and Zn^2+^ in erythritol biosynthesis by the cells of *Y. lipolytica*, the activity of erythrose reductase was measured in the samples from bioreactor cultivations—the control culture as well as cultures supplemented with minerals (Table 1). The samples used in this experiment were obtained after 24 h of cultivation (Fig. 2). The correlation of erythrose reductase activity with the erythritol biosynthesis parameters was presented in Fig. 3. The erythritol production parameters were improved in accordance to the increased activity of erythrose reductase, caused by culture supplementation with minerals. The most pronounced effect on the erythrose reductase activity was observed upon Mn^2+^ addition to the culture, where activity of the enzyme increased 1.3-fold compared to the control and reached 24.9 U g^−1^ of DW. It is worthy of notice that the high activity of the enzyme corresponded to the best production parameters achieved in that culture. The stimulatory effect was observed also when Cu^2+^ and Zn^2+^ ions were added to the culture resulting in the erythrose reductase activity at 22.7 and 23.0 U g^−1^ of DW, respectively. A similar effect of Cu^2+^ on the activity of erythrose reductase was reported for erythritol biosynthesis by yeast from the *Torula* genus [8, 25]. Furthermore, it was speculated that mechanisms of Cu^2+^ effect promotion were mediated by the inhibition of fumarate which is a strong inhibitor of erythrose reductase. Although no stimulatory effect of Mn^2+^ on the enzyme activity was observed for *Torula* sp., the improvement of erythritol biosynthesis in the presence of these ions was explained by increased cell permeability that enabled outflow of intracellular erythritol [8].

The effect of trace elements on the activity of other *Y. lipolytica* enzymes was reported in the literature. When yeast were cultivated in ethanol-containing media, zinc was found to increase the activity of the enzyme responsible for the first step of ethanol utilization—alcohol dehydrogenase, a zinc-containing enzyme [28]. Moreover, it was demonstrated that high iron concentrations positively affected the activity of iron-dependent enzymes, aconitate hydratase, and catalase, resulting in increased production of citric and isocitric acids from ethanol [28, 29]. Similar effect of iron on the activity of aconitate hydratase was observed during citric acid biosynthesis from rapeseed oil [31]. Different effects of the trace elements on the processes with *Y. lipolytica* might be explained by the differences between metabolic pathways involved in the biosynthesis of erythritol and tricarboxylic acid cycle metabolites.

## Conclusions

The impact of minerals on the production of erythritol and activity of erythrose reductase in biosynthesis by *Y. lipolytica* when grown on glycerol was reported for the first time. The strain, Wratislavia K1, exhibited high tolerance to divalent copper, iron, manganese, and zinc ions, as yeast growth was not reduced after their addition. The best results were observed after supplementation with MnSO_4_·7H_2_O (25 mg l^−1^) where erythritol production reached 47.1 g l^−1^ with the yield of 0.47 g g^−1^ and volumetric productivity of 0.87 g l^−1^ h^−1^. The improved erythritol biosynthesis in that culture was correlated with an enhanced activity of erythrose reductase to 24.9 U g^−1^ of DW, i.e., about 1.3 times higher activity than in the control culture. Worthy of notice is that the proposed biosynthesis was characterized by a very low formation of by-products, which is a beneficial feature in the industrial application. Moreover, attention should be paid to the feasibility of applying glycerol as the sole substrate in the process, as it is advantageous from both the environmental and the economic perspective when compared with glucose.

## Abbreviations

DW: dry weight of biomass
GLY: glycerol
X: biomass
ERY: erythritol
MAN: mannitol
Y_ERY_: erythritol yield (g of produced erythritol per g of used substrate)
Q_ERY_: erythritol volumetric productivity
q_ERY_: erythritol specific production rate
